# Development and Differentiation in Monobodies Based on the Fibronectin Type 3 Domain

**DOI:** 10.3390/cells9030610

**Published:** 2020-03-04

**Authors:** Peter G. Chandler, Ashley M. Buckle

**Affiliations:** Department of Biochemistry and Molecular Biology, Biomedicine Discovery Institute, Monash University, Clayton 3800, Australia; ashley.buckle@monash.edu

**Keywords:** adnectin, biosensor, fibronectin, monobody, non-antibody scaffold, therapeutic

## Abstract

As a non-antibody scaffold, monobodies based on the fibronectin type III (FN3) domain overcome antibody size and complexity while maintaining analogous binding loops. However, antibodies and their derivatives remain the gold standard for the design of new therapeutics. In response, clinical-stage therapeutic proteins based on the FN3 domain are beginning to use native fibronectin function as a point of differentiation. The small and simple structure of monomeric monobodies confers increased tissue distribution and reduced half-life, whilst the absence of disulphide bonds improves stability in cytosolic environments. Where multi-specificity is challenging with an antibody format that is prone to mis-pairing between chains, multiple FN3 domains in the fibronectin assembly already interact with a large number of molecules. As such, multiple monobodies engineered for interaction with therapeutic targets are being combined in a similar beads-on-a-string assembly which improves both efficacy and pharmacokinetics. Furthermore, full length fibronectin is able to fold into multiple conformations as part of its natural function and a greater understanding of how mechanical forces allow for the transition between states will lead to advanced applications that truly differentiate the FN3 domain as a therapeutic scaffold.

## 1. Introduction

### 1.1. Monobodies Based on FN3

**Figure 1:**
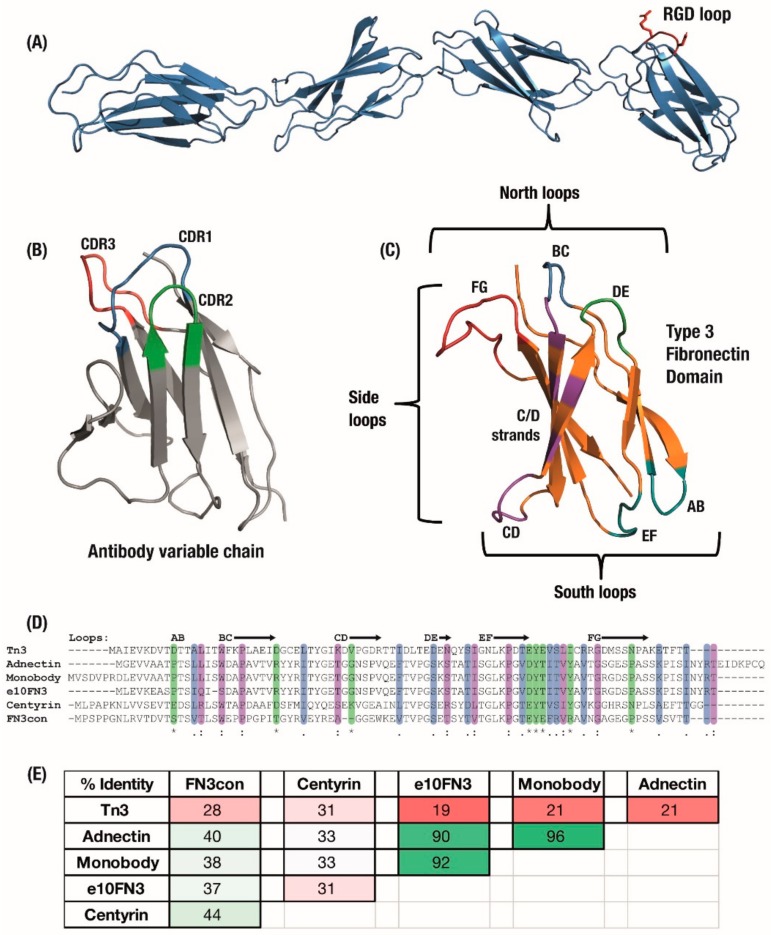
(**A**) The Type 3 Fibronectin domains 7 to 10 from human fibronectin with the original RGD binding sequence highlighted in red. (**B**) Antibody domains use a set of three hypervariable binding loops to form a complementary region to a target binding site. (**C**) Fibronectin type III (FN3) domains have a comparable set of analogous loops which can be engineered for similar binding function, as well as an expanded binding footprint in the side and ‘south’ loops. Six derivatives of the FN3 domain under development have similar size and structure but can vary widely in (**D**) amino acid sequence, sharing only the F-Strand sequence across the domains, which leads to (**E**) a large variation in overall sequence identity between derivatives. Colouring: (**D**) Sequence alignment: */green—identical amino-acid,:/purple—strongly similar,/blue—weakly similar.; (**E**) Sequence pairwise identity matrix: green—highly identical sequences, pale green—strongly identical sequences, pink—weakly identical, red—low identical amino-acid matches.

The Fibronectin Type 3 domain (FN3) is an established scaffold for developing non-antibody binding domains [1,2,3,4]. Consisting of an Ig fold and retaining Complementarity Determining Region (CDR)-like structures of an antibody (Figure 1B,C) without disulphides, its structural simplicity offers advantages for the engineering of advanced non-antibody functions [2,4,5]. The native context of the fibronectin domain in the extracellular matrix also results in potential reductions in immunogenicity, due to its abundance in the human body [4,6]. This structural similarity between the FN3 and antibody variable domains allows established knowledge of beta-sandwich binding domains to be applied to a novel non-immunoglobin scaffold. However, antibodies remain the industry and clinical gold standard, due to the established knowledge-base on antibody development, proven clinical effectiveness and decades of investment in optimising antibody CMC (Chemistry, Manufacturing and Controls). In response, clinical therapeutic proteins based on the FN3 scaffold are beginning to use native fibronectin function as a point of differentiation.

### 1.2. The Fibronectin Assembly Offers Functional Differentiation from Antibodies

A challenge for the diverse range of non-antibody protein scaffolds is to exploit native characteristics and produce tailored applications [1]. The advantage of a tailored approach is that wildtype functions of each domain can be leveraged to overcome limitations of the traditional immunoglobulin fold, such as the modular construction of DARPins [7], convex binding surfaces of Affibodies [8] or protease targeting in Kunitz domains [9]. For FN3 domains, their native functions can be exploited to produce novel binders and to design context-sensitive drugs or biosensors [10].

Full-length fibronectin includes multiple repeats of the Type 3 Fibronectin (FN3) domain, combined with type 1 and 2 fibronectin domains and others, with each FN3 domain naturally associating with multiple targets involved in forming the extracellular matrix. This association was originally seen to occur via a conserved ‘RGD’ amino-acid motif in the FG loop of some FN3 domains (Figure 1A); as such, the initial focus for engineering a binding surface centred on the CDR-like BC, DE and FG ‘north’ loops. These loops have been engineered with different lengths and amino-acid diversities ranging from binary Tyr/Ser [11] to mimicking antibody CDR composition [12]. It is now understood that FN3 domains incorporate multiple ‘association zones’ across the entire beta-sheet structure [13] (Figure 1C), which has led to development of the ‘side’ surface including the FG loop, F and G strands to produce a concave binding surface [3,14,15] and the ‘south’ AB, CD and EF loops [5,16,17]. In line with its natural function, most of the surface of the FN3 molecule is amenable to evolution for association with target proteins and bivalent monobodies have been developed which make concurrent use of separate binding surfaces [18].

In contrast to the rigidity in antibody beta-sheets imposed by internal disulphides, the mode of interaction between fibronectin and its targets involves a combination of loop flexibility and beta-sheet dynamics that allows FN3 domains to adopt multiple optimal binding conformations [13,19]. Additionally, without the large tertiary structure of antibodies, a single monobody has the potential to interact with smaller or less accessible binding pockets on a target molecule [20]. Moreover, internal ‘cryptic’ binding surfaces offer new modes of interaction not available to most binding scaffolds [21], and where multi-specificity is challenging with an antibody format that is prone to mis-pairing of light and heavy chains, FN3 domains in the fibronectin assembly already interact with a large number of molecules through a beads-on-a-string fusion (Figure 1A) [19]. As such, multiple monobodies engineered for interaction with unique therapeutic targets have been combined in a similar modular assembly which improves both efficacy and pharmacokinetics [22,23,24].

### 1.3. Modern FN3 Derivatives in Development

The Type 3 Fibronectin scaffold has been developed into a range of monobody derivatives (Figure 1C), similar to the multiple variations of the antibody scaffold. Although these monobody derivatives maintain a similar Type 3 Fibronectin structure [25], they vary widely in their amino-acid sequences, optimisation for expression and thermal stability. The sequences of six major variants have been summarised in Figure 1D,E. These began with the original ‘Monobodies’ [2,26], which initially focused on a library of full BC and shortened FG loops selected by phage display. Clinical stage Adnectin monobodies (from Adnexus, now under Bristol–Myers Squib), were developed from the FN3 scaffold with libraries of full-length BC, DE and FG loops selected by mRNA display [5]. Another monobody derivative comes from a wild-type FN3 scaffold selected for loops which bound IκBα but which resulted in a substantial trade-off in expression levels; as a result, the e10-FN3s were engineered which featured an altered AB loop (“AATPTSLLI” changed to “EASPTSLIQ”) and rescued expression levels [27,28]. The e10-FN3s are also being developed as ‘intrabodies’ under the names FingRs—Fibronectin intrabodies generated with mRNA display [29] or FANGS—Fibronectin Antibody-mimetic Nicotinic acetylcholine receptor Generated ligands [30].

An initial alternative approach was to make use of similar FN3 domains from Tenascin instead of Fibronectin, which was then stabilised largely with the introduction of a cysteine bond between the C and E beta-strands [21]. This Tenascin-FN3 derived monobody is now under clinical development as ‘Tn3′ by Viela Bio (spun out from AstraZeneca’s Medimmune) [31,32]. Finally, in order to design more robust monobodies with substantially higher initial stabilities, the consensus bioinformatic technique [33] has been applied to generate Aro Therapeutic’s Centyrins from a consensus sequence of 14 FN3 domains (spun out from Janssen) [34,35] and the hyper-stable “Consensus FN3” FN3Con domain [36] from a consensus of 2123 FN3 sequences. As a result of this differentiation, these synthetic domains share the parent FN3 fold but feature varying sequence similarity (Figure 1D,E).

The current field of monobody development also benefits from mature technologies that can deliver binding reagents on demand [37] through yeast surface display [38], phage display [39] and mRNA display [40]. The scaffold is also viable for grafting and evolution from single peptide ‘anchors’ [18,41,42,43] or CDR-like loop grafting between FN3 scaffolds [44,45,46].

### 1.4. Learning from the First Clinical Monobodies

The Adnectin-anti-VEGFR2 ‘CT-322′ was the first of the monobody family to reach clinical trials [17,47]. However, the Adnectin showed a lack of required efficacy in phase 2 clinical trials treating recurrent glioblastoma [48], which was suspected to be caused by a lack of clinical effect from inhibiting VEGFR2 (vascular endothelial growth factor receptor-2) activity (possibly related to broader failures in treatment through the VEGF pathway [49] or from issues with pharmacokinetics of the small Adnectin scaffold).

In spite of this clinical failure, the Adnectin-anti-VEGFR2 has become a model protein for development of technologies supporting the monobody scaffold, with recent studies aiming to enhance the Adenctin’s pharmacokinetic properties through PASylation [50] or improve CMC through bacterial expression [51]. The wider lesson learnt from this first clinical attempt was primarily to select targets and applications that differentiate the FN3 scaffold instead of following established antibody methodologies, such as the re-purposing of the Adnectin-anti-VEGFR2 as an ultrasound imaging agent [52] or in CAR-T (Chimeric Antigen Receptor-T Cell) formats [53].

## 2. Monomeric Monobodies in Therapeutics and Diagnostics

The small size (~10 kDa molecular weight) of individual FN3 domains confers increased tissue distribution and reduced half-life, whilst the absence of disulphide bonds improves stability in cytosolic environments and facilitates the introduction of non-native cysteines. The applications of monobodies in fundamental science discovery have been covered elsewhere [3], including for blocking of multiple sites on a target protein [54], altering activity through allosteric modulation [55], and as crystal chaperones [56]. However, more recently the monobody domain has found unique applications in the fields of therapeutics and diagnostics.

### 2.1. Delivery Agents

Early Antibody Drug Conjugates (ADCs) were limited by properties of the antibody scaffold. The high molecular weight of intact antibodies prohibited distribution throughout a solid tumour and instead these antibodies and their toxic conjugates remained in blood circulation due to recycling processes that target the Fc region and glycosylation [57]. This was estimated to result in only 0.01% of total ADCs deposited into tumour cells [57], which has resulted in only a few approved ADC biologics despite more than 60 ADCs entering clinical trials [58]. The first clinical Adnectin was targeted towards VEGFR2 as it was thought to offer a competitive advantage to full size anti-VEGF antibodies, due to the ability of smaller domains to better penetrate a solid tumour. While the effectiveness of that treatment did not eventuate, it led to the development of drug-delivery approaches that utilised the greater tissue distribution of small monobodies.

Monobody domains have been developed as part of the latest wave of ADCs, as their smaller physical size results in improved diffusion and extravasation that enables faster tumour penetration [59]. Small non-antibody formats also offer advantages such as fine-tuning of affinity to low nanomolar levels, enabling them to pass through the outer layers of tumour cells and avoid a ‘barrier effect’ [59]. The absence of cysteines in FN3 domains allows for the introduction of a single conjugation-compatible cysteine for site-specific loading with drugs, thus avoiding potential disulphide-mediated aggregation [60,61]. This feature is critical as cytotoxic drugs can be disruptive to protein structure due to their hydrophobic nature, and small protein scaffolds require well-controlled conjugation to ensure resistance to aggregation [35,57]. In practice, these advantages have been demonstrated with a glypican-3 specific Adnectin monobody domain loaded with a cytotoxic derivative of tubulysin [58]. Complete conjugation and a drug to antibody ratio (DAR) of 1:1 was achieved with an introduced cysteine at the monobody C-terminus. Due to its small size, the Adnectin was cleared from the murine and cynomologous bloodstream within 30 min and 2 h, respectively, via renal filtration. As well as limiting the exposure to off-target tissues, renal clearance offered another advantage as clearance by liver metabolism will often cause off-target release of cytotoxic conjugates. In fact, this study found that a single dosage of the ADC was able to inhibit tumour grown for up to 2 weeks, demonstrating clinical effectiveness regardless of the short circulating half-life.

**Figure 2:**
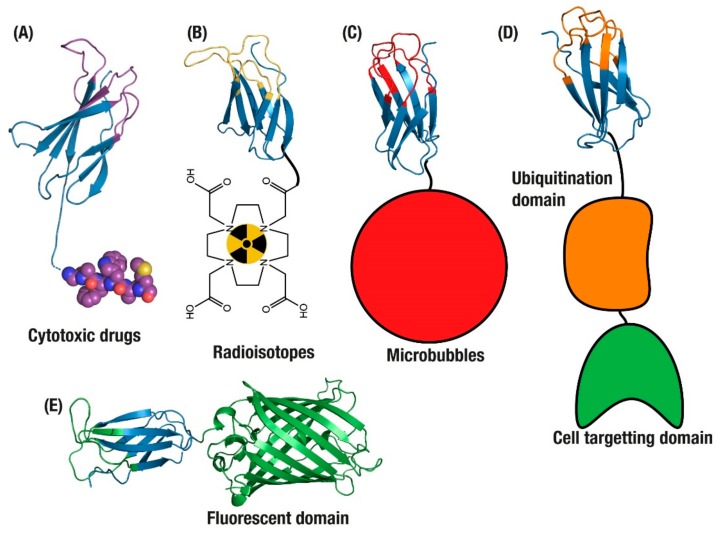
Applications of monomeric antibody domains. (**A**) Antibody Drug Conjugate (ADC) drug delivery [58]. Tumour imaging with monobodies conjugated to (**B**) radioisotopes [62] and (**C**) microbubbles [52]. (**D**) Targeted degradation of endogenous intracellular proteins [63] and (**E**) targeted intracellular fluorescence reporters for endogenous proteins [64].

The ADC applications of monobodies and other small non-antibody scaffolds, however, are disadvantaged by the absence of certain features present in antibodies. Notably, cysteine conjugation in an antibody results in an average DAR of 4:1, where a single introduced cysteine on a monobody will result in a DAR of 1:1 (Figure 2A). This may not be enough to deliver a completely cytotoxic dose, despite the observation that DARs above 4 see reduced returns in efficacy [57]. Secondly, as non-antibody scaffolds are characterised by fast clearance from the bloodstream resulting in a short circulating half-life, solid tumours may never experience complete saturation with a conjugated drug [59].

### 2.2. Biosensors

Given these challenges, design has shifted focus instead to biosensor applications, such as combining radioisotopes with tumour cell targeting binders. In one application, Adnectins were generated for non-invasive imaging of PD-L1 with radioisotopes (Figure 2B) [65]. PD-L1 is an immune suppression ligand that is overexpressed on the surface of tumours, making it a selective target for the imaging of tumours before and during treatment [66]. However, as PD-L1 is expressed on most cells as part of normal suppression of auto-immune response, the use of antibodies to deliver radioisotopes for imaging is limited since the long circulation time and off-target binding of antibodies results in considerable background signals and reduced tumour penetration while inducing systemic radiation burden to patients [66].

Here the small monobody size overcomes some limitations of antibodies, resulting in greater tumour penetration and limited circulation [65]. These advantages were validated as part of human safety studies with an ^18^F loaded Adnectin, where imaging could be completed on the same day as injection and antibody-based reagents may take several days of radiation burden to clear non-specific binding [67,68]. This was further confirmed after loading the Adnectin with ^64^Cu where the monobody was able to provide same-day PET visualisation of tumour cells, resulting in an increase in the monobody uptake in tumours over 24 h post-injection [62,69]. This concept has been taken even further with a Centyrin domain evolved to bind EGFR and conjugated with a fluorescent dye, which was then applied to guide surgical removal of tumour cells [70].

### 2.3. Intracellular Applications

A core advantage offered by the monobody domain is the absence of a disulphide bond that antibodies may require for stability; this is an advantage, specifically in the reducing cytosolic environment.

This feature has been exploited to explore protein function within cells, as current immunohistochemistry methods of detecting proteins inside cells often require a cell to be fixed, permeabilised and then soaked with antibodies, which is highly disruptive to the natural environment within a cell. In addition, genetically tagging target proteins with GFP-based fluorescence alters the expression and location of endogenous proteins. In developing approaches to monitor endogenous proteins in the cell, the FN3 domain was applied as an internal reporting agent of endogenous proteins in live cells (Figure 2E) [29]. This application was attempted in neuronal cells, where visualising endogenous proteins provided a means of understanding neuronal structure and function. These intracellular monobodies were then applied to visualise dynamics in living cells without altering expression or location of the endogenous targets, and was able to follow the movement of an intracellular vesicle at 7 μm/s [29]. This work was taken even further with the development of state-selective intracellular binders of Ras, a commonly mutated oncoprotein in tumours with poor prognoses [64]. The anti-RAS monobodies specifically targeted K- and H-Ras, two variants that are the most commonly mutated genes in human tumours, and were demonstrated to be highly specific tools for monitoring the conformational or mutational state of RAS proteins while also potentially modulating their signalling pathways [64].

Alteration of signalling pathways was also applied through steric inhibition on target interactions, as the monobody domain can be used intracellularly to understand and modify protein interaction networks. Although complete mutational knockouts are important for understanding the importance of a protein to its network, a single protein may interact with an average of four partners in multiple pathways which are all disrupted in a protein knockout [71]. To overcome this, FN3 domains evolved to bind proteins in the Wnt signalling pathway were expressed endogenously to block intermolecular interactions of individual domains within a quaternary protein assembly. This approach allowed signalling proteins to be annotated by individual domain [71] and was taken into mouse models to target WDR5, a component of mixed lineage leukemia, to effectively suppress leukemogenesis [72].

Further to blocking activity in endogenous intracellular proteins, the monobody domain has been combined with ubiquitin-tagging domains to selectively induce degradation of those intracellular proteins. By fusing to domains that confer degradation, such as the Von Hippel–Lindau oncoprotein suppressor (VHL) or E3 ubiquitin ligases (Figure 2D), monobodies can deliver degradation signals directly to endogenous proteins within a target cell [63,73]. This specificity can then be improved by adding multiple monobodies or cell targeting domains [63,74].

This mix-and-match approach of combining monobodies mimics the natural assembly of FN3 domains in fibronectin as a beads-on-a-chain pattern with each FN3 domain interacting with a unique set of targets in the extracellular matrix [25]. This represents a unique differentiating factor from antibodies, where bispecific antibodies and scFv combinations must be carefully designed to reduce domain mis-assembly [22].

## 3. Multidomain Monobodies

The small size and short half-life of the monobody scaffold has been exploited in imaging and delivery applications. However, these properties are limitations to developing the scaffold as a next-generation therapeutic platform. Whereas antibodies have extended circulation lifetime due to natural functions of recycling via their Fc domains and glycosylation, monobodies must be engineered to match these critical clinical features. To date, most non-antibody biologics have made use of polyethylene glycol (PEG) to increase their size, reduce renal filtration and lengthen circulating half-life. This polymer is produced as a mixture of molecular weights with high polydispersity, is not amenable to further chemical functionalization and is known to produce formaldehyde upon degradation. Alternatively, a straightforward application of fibronectin-like modular design is to increase circulating half-life and thus improve pharmacokinetic properties via fusion with non-monobody domains [75].

### 3.1. Fusion to Extend Half-life

**Figure 3:**
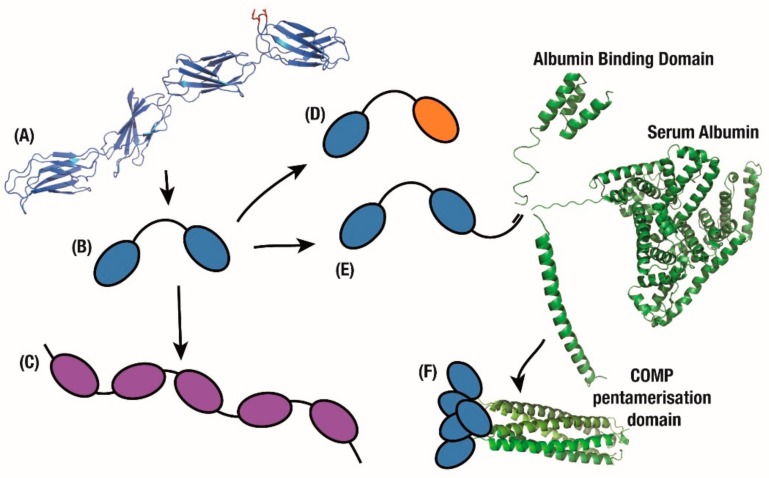
Multimeric applications. (**A**) FN3 domains on the fibronectin chain exhibit binding to multiple partners. Mimicking this beads-on-a-string approach quickly produces (**B**) bivalent, (**C**) tetravalent and (**D**) bispecific monobody constructs. (**E**) Furthering this fusion approach, tandem monobodies are fused with domains which confer longer circulating half-life or (**F**) greater avidity.

In order to maintain the structural simplicity of monobody constructs, multiple non-Fc fusions have been attempted. By conjugating to various mutants of the Albumin Binding Domain (ABD) from Protein G that have a gradient of affinity to Serum Albumin (Figure 3E), the half-life of a monobody was increased from 5 min to between 8 and 80 h. Since the ABD can be engineered to fine-tune monobody release from circulating Serum Albumin and thus half-life, this represents another advantage over the recycling of antibody Fc domains which occurs at a single, controlled rate [76]. To further improve the biophysical properties of this assembly, a thermostable version with both a consensus-stabilised monobody and a consensus-designed ABD [77], was used for prophylactic treatment of *Staphylococcus aureus* infections [78].

The small and simple structure of a monobody is attractive given the importance of reducing manufacturing costs for biologics, if only to lessen this burden in the early stages of research translation. To this end, an Adnectin monobody was raised to mimic the effect of approved antibodies Evolocumab and Alirocumab on the Coronary Heart Disease target PCSK9 (Proprotein convertase subtilisin kexin 9). These antibodies showed a reduction of LDL Cholesterol over 60% by blocking PCSK9-led degradation of the LDL-C Receptor [79]. However, they were originally released at a price of ~$14,542 USD per year, which was analysed as well over a reasonable yearly price of ~$4215 USD for their quality of life benefit [80]. As a result, a range of inhibitors against PCSK9 are under development, including monobodies with their reduced manufacturing-cost burden.

Conjugation of PEG to an Adnectin monobody that inhibited PCSK9 activity resulted in a molecule that showed a marked cholesterol decrease in pre-clinical models while extending circulation time [81]. Unfortunately in humans this construct reduced LDL-C by only 47% at the highest intravenous dose, or by 48% as a subcutaneous dose in combination with statins [82], in stark contrast to the efficacy of approved antibodies such as Evolocumab which reduce cholesterol by 60% or more with similar doses [79]. Alongside this work, an affibody non-antibody domain which targeted PCSK9 was fused with serum albumin for longer half-life [79,83]. In a similar application, and to further improve efficacy, a serum albumin domain was also fused to the Adnectin-anti-PCSK9 construct to improve circulation half-life to match that of antibodies (Figure 3E) [79]. This resulted in comparable pharmacokinetics and cholesterol reduction to evolocumab [84], with a phase 2 study presenting similar efficacy with a 70% decrease in cholesterol 8 weeks after treatment, closely mimicking the effects of comparable antibodies [85].

### 3.2. Combining Monobodies with Antibodies

**Figure 4:**
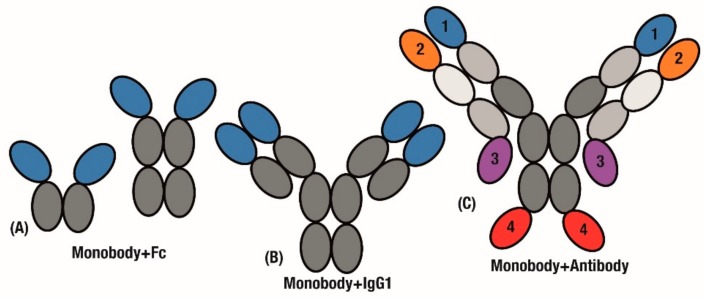
Applying monobody advantages to the antibody scaffold. Monobodies are fused with antibody fragments to extend half-life and generate bivalency. Replacing either (**A**) Antigen-binding fragments or (**B**) individual variable domains. (**C**) mAbtyrins extend this combination through developing by specifics by fusing monobodies to 1 of 4 positions on the C- or N-terminal ends of either chain.

The final application of this modular assembly aims to combine useful antibody characteristics such as Fc recycling or bivalency, and build on them to generate improved therapeutics. For example, monobodies have been used in place of scFv domains (Figure 4A), which can be prone to mis-assembly. After classical antibody inhibitors had failed to show an effect on muscle growth in Duchenne Muscular Dystrophy (DMD) [86], an Adnectin monobody targeting myostatin was fused with an IgG1-Fc domain to produce a clinical inhibitor [87]. Interestingly, PEGylation was again found to be an inferior method of half-life extension following discovery work identifying Fc fusion as a superior tag [75]. This anti-myostatin fusion was shown to increase skeletal growth in pre-clinical models of DMD [88] and healthy humans [89] and is now in an ongoing phase 2 investigation for treatment of DMD [90].

In a further extension of this design, Tn3 monobodies were fused to replace either the entire scFv region or individual variable domains (Figure 4A,B). The added avidity of a tetravalent molecule (Figure 4B) resulted in the Tn3 and IgG fusion displaying improved effectiveness over the bivalent Tn3 and Fc fusion construct (Figure 4A) [91].

Further to replacing scFv domains in antibodies, significant challenges in generating bispecific antibodies [92] can be overcome by simply fusing monobody domains to an established antibody. A key case study for bispecific antibodies is in activating cooperative receptors. In order to drive activation of Tumour Necrosis Factor (TNF) pathways, antibodies that target the TNF receptor OX40 were fused with monobodies that engage the Fc-γRIIB receptor to further stimulate anti-tumour immunity. These Centyrin monobodies were fused to the C- and N-terminal of both heavy and light chains in the antibody construct to generate variants of the ‘mAbtyrin’ construct (Figure 4C), with monobodies at all of the positions resulting in bispecificity [93].

Monobodies have also been designed to replace scFvs in other contexts including next-generation CAR-T therapies. Where antibodies and their resulting scFvs can be difficult to generate and fine-tune for affinity, monobody evolution systems readily generate binders which can be integrated into CAR-T systems [94]. Adnectin monobodies were introduced into CAR-T systems to target tumours overexpressing VEGFR2 or EGFR [53,94], and through reducing affinity to EGFR the CAR-T cells were engineered to only reach effective concentrations at tumour cells which drastically over-express the target receptor.

To further improve on this fusion approach, Centyrin monobodies were integrated into a CARTyrin expression system which combines selection markers and a safety self-killing switch into a simple mRNA and plasmid DNA transcription system [95]. CARTyrins have been generated for targeting Multiple Myeloma [95] showing effective elimination of tumours in head-to-head trials against scFv-based CAR-T therapies, with targeting of prostate cancer also raised as a possible indication for treatment [96]. The Multiple Myeloma CARTyrin was presented as passing safety and efficacy endpoints in a phase 1 trial [97]. This resulted in the design of a phase 2 study [98], which is currently ongoing (PRIME; NCT03288493).

### 3.3. Multi-Valent and Multi-Specific Monobodies

A key limitation in developing antibodies, as discussed earlier, is uncontrolled mispairing between heavy and light chains. This is exacerbated in bispecific antibodies, where only one of many possible interdomain pairings will yield a viable multivalent molecule [22] with the best reported results including an 85% correct bispecific pairing [99]. In stark contrast, mimicking the modular physiological state where multiple fused FN3 domains act independently can result in monobody chains with multi-valency or multi-specificity [75]. This mix-and-match approach of combining monobody domains mimics the natural assembly of FN3 domains within fibronectin as a beads-on-a-chain pattern (Figure 3A), with each FN3 domain interacting with a unique partner in the extracellular matrix [25]. This represents a unique differentiating factor from antibodies, where bispecific antibodies and scFv combinations must be carefully designed to reduce domain mispairing and antibody mis-assembly [22,99].

Monobodies have previously been co-administered as a potential combination therapy [100]. However, combination dosages rely on drugs reaching target tissues through separate pathways and combination through fusion is an important next step for improving pharmacodynamics and targeting. As a result, bispecific monobody fusions have been generated for increased avidity and selectivity against tumour tissues by targeting over-expressed receptors IFG-IR and EGFR in the case of Adnectins [101].

An early example of this development is an Adnectin monobody which was evolved to target CD4, a target receptor for HIV entrance into cells, and thus sterically block HIV internalisation via CD4. The major advantage was that, in contrast to an antibody, the monobody could block the HIV interaction epitope without blocking the natural function of the CD4 receptor [102]. In order to improve upon this mechanism, the anti-CD4 monobody was then fused to an anti-gp41 monobody which binds a surface protein on the HIV capsid (Figure 3D). This combination was then able to use the HIV virus to deliver prophylactic biologics to the site of infection [103]. Important to this engineering is to learn from the lessons of designing multi-valent antibody constructs [104] where the length of amino acid linkers between each domain must be considered to ensure tandem monobodies are able to sterically access their targets [103].

A key improvement in moving away from the antibody scaffold is avoiding immunoreactive properties intrinsic to natural antibody function. For example, the CD40 receptor is involved in the activation of immune cells that produce autoantibodies and is a key route of pathology in autoimmune disease [105]. Biologics have been developed to block activity of the CD40 ligand (CD40L) in order to reduce reactivity in autoimmune diseases, however antibody biologics mimic the actual autoimmune antibodies generated as part of the disease [105]. In such an application, a Tn3 monobody was evolved to bind CD40L as a crystallisation chaperone [106], but was later re-engineered to mask cells at risk of autoimmune reactions [107]. A tandem anti-CD40L monobody construct fused with serum albumin (Figure 3B) was then designed for clinical applications under the names MEDI4920 then VIB4920 [105]. This construct was devoid of the reactive Fc region present in autoantibodies and reduced immunostimulatory action over time in humans, resulting in increased safety and reduced risk of thromboembolic events [108,109].

While multi-specificity is an important design aim in therapeutics, antibody multivalency is a feature that the monobody domain can improve upon. Medimmune was able to take this approach of multivalent Tn3 monobody fusions further with a range of mono- to octa-valent constructs (Figure 3C) to generate superagonists to the TNF-related apoptosis-inducing ligand receptor 2 (TRAILR2). Increasing the number of receptors activated by the monobody fusion substantially improved apoptotic signalling, up to a limit of 4 to 6 domains, after which all of the local cell surface receptors were presumably activated [91]. This improvement over the bivalency of antibodies was also recently demonstrated as a tumour killing agent in multiple pre-clinical tumour models of breast cancer [110]. The approach of increasing avidity by increasing the number of monobody molecules has been conceptually taken further with a polyvalent construct (Figure 3F). In this case, each monobody was fused to a COMP pentamerisation domain to create a polyvalent construct [111].

Where multi-specificity is challenging with an antibody format that is prone to domain swapping, FN3 domains in the modular fibronectin assembly are already multivalent and multispecific. By utilising this native assembly of FN3 domains in fibronectins, multiple approaches can be designed that overcome head-to-head limitations against antibody half-life and circulation. More critically, modular combinations can easily match antibody multispecificity or multivalency to differentiate the monobody scaffold in a way that surpasses the limitations present in immunoglobulin domains. In the field, this has developed in fusions like the tandem bi-valent Tn3 monobody fused to a Serum Albumin domain [105] or tandem bi-specific Adnectins that utilise binding to HIV proteins to deliver prophylactic domains to the site of HIV infection [103], with many more multivalent or multispecific approaches to be developed.

## 4. Future Applications of the Monobody Scaffold

Full length fibronectin is able to fold into multiple conformations as part of its natural function. The fibronectin assembly is involved in the formation of the extracellular matrix (ECM), and thus has an internal mechanism for the transition from soluble monomeric conformations to larger oligomers and finally insoluble ECM [19]. This results from a conformational change where beta strands are exchanged between modules, which opens a hydrophobic binding site and promotes aggregation [112,113]. This conformational change is kinetically controlled [32] and is triggered in reaction to environmental stresses [25].

Domain swapping is usually removed through stabilisation of the monomeric fold, since any tendency for oligomerisation complicates developability [114], for example in the use of the scaffold for therapeutic applications. Similar strand exchange has been studied extensively in other IgG fold domains [115,116,117] and may be related to a wider problem of domain swapping and higher order structure formation in biologic therapeutics [118]. For example, there is recent evidence that pH governs the integrity of protein higher order structure, which can be critical for the application of novel biotherapeutics [119].

Enhancing the thermostability of FN3 domains may reduce or even eliminate strand exchange function, however in practice mobility of domain-swapping beta-strands has been observed in structural studies of thermostable Centyrins [120,121], molecular dynamics simulations of multiple monobody domains, including the thermostable FN3Con [36] and through assaying the unfolding of Adnectins [122]. In the course of developing monobodies, oligomer formation has been used as a negative selection factor [58,123] and structural analysis of higher order oligomers has brought about design rules around monobody loop length and sequence make-up [120,121].

However, developing a greater understanding of the conformational flexibility of fibronectin and how mechanical forces allow for the transition between active and inactive states provides a route to context sensitive applications. Further, where inter-domain exchange may alter the presentation of a primary interface for binding, it may also create a unique secondary interface that is also active and context-specific [124]. Applications of this concept are still largely under construction, however, two unique outcomes of interest are under development in generating monobodies that are pH responsive or act as biosensors.

### 4.1. Exploiting Responses to pH Change

A well-studied feature of the fibronectin aggregation mechanism is conformational change in reaction to environmental conditions. One environmental change of interest is that the mechanical stability of the 10^th^ FN3 domain has been shown to be responsive to changes in pH [112]. This includes an improved ability to refold [125], as well as a small pH reduction resulting in large functional changes in vivo [126].

pH responsiveness has previously been applied in other therapeutic domains to optimise release from various parts of the low-pH endosomal degradation pathway. As high-affinity binding often results in antibodies following their targets into endosomes, anti-PCSK9 antibodies have been evolved to almost completely lose their affinity in low-pH to promote recycling back into circulation [127]. Instead of engineering CDRs, Fc domains have been developed for higher affinity to the Fc-Receptor at low-pH, which allows antibodies to be rescued from endosomes then released into the cytosol [128]. In a final example of exploiting pH changes, endosomes have been targeted with drug-delivery nanoparticles that chaperone drugs into degradation compartments, then release the molecules at low-pH [129].

In a similar application to the anti-PCSK9 antibodies, monobody CDRs were evolved for reduction in affinity at low pH. Although this design was able to yield monobodies with a 10-fold difference in affinity between pH 5.5 and 7.4, most of the mutants suffered a trade-off in stability to achieve this function and did not survive the shift from complex mammalian expression used in directed evolution to the more crude *Escherichia coli* expression systems [10]. A key limitation of this approach was the application of antibody approaches by CDR engineering, where an applied understanding of FN3 conformational flexibility could have yielded novel approaches. An application of this understanding is in using monobodies as biosensors which undergo stand-exchange to become active FRET (Forster Resonance Energy Transfer) sensors [21].

### 4.2. Native Strand-Exchange Function

The inclusion of a signalling moiety into proteins to create binding sensors remains a substantial challenge. Previously, changes in conformation have been exploited for FRET signalling [130], however conformational change is not available in antibody folds. Additionally, the conformational change of 30–70Å required for an optimal FRET signal is generally not possible for small binding proteins. One example that circumvents this is the incorporation of fluorescent non-natural amino acids that are sensitive to environmental changes such as solvation of a binding surface [131].

**Figure 5:**
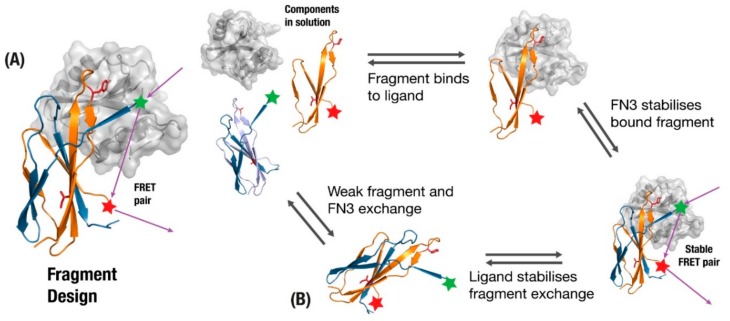
(**A**) monobody fragment is generated from fragments involved in binding (orange), where the original monobody undergoes loss-of-function mutations in residues to remove affinity to the target and loss-of-stability mutations to disrupt beta-sheet packing (Red). (**B**) The presence of ligand and fragment allow a fragment-exchange complex to form that brings a Forster Resonance Energy Transfer (FRET) pair together, providing biosensors for the presence of a ligand.

One of the best established examples of incorporating fibronectin function into monobodies is the FREX biosensor design, which exploits the tendency of wildtype fibronectin to kinetically exchange fragments between multiple FN3 domains [21]. A beta-strand involved in the binding interface undergoes mutations to reduce affinity and packing (Figure 5A) such that when a fragment with the original sequence is added in solution it will preferentially trade the new fragment with its sub-optimal strand. The full monobody and new fragment are then given complementary pieces of the FRET system, which activate upon fragment-exchange. Two key features of this design are that the fragment exchange will only preferentially take place if the binding target is present (Figure 5B), and the FRET tagged components will be effectively out of range until the target protein brings them together (Figure 5B). Although the authors describe this as a scaffold-independent application [132], scaffolds based on proteins that are prone to inter-domain exchange should be more amenable to this approach.

## 5. Conclusions

A major challenge for the developing field of antibody-agnostic binding proteins is to meaningfully differentiate from the current antibody gold-standard. However, it is important to understand the historical background to antibody development. Antibodies were originally developed in part because natural immune systems could be harnessed to non-rationally develop highly specific, high affinity binders of therapeutic targets. Now that the process of directed evolution has been largely abstracted into laboratory processes [38,133,134], the concept of harnessing natural function to generate new therapeutic modalities can be applied to a wider field of scaffolds. Where the antibody domain is limited in its capacity for multi-specificity, multi-valency and context-specific activity, FN3 domains naturally present these functions. As a result, exploitation of native fibronectin functions will lead to advanced applications that truly differentiate the monobody as a therapeutic scaffold.

## Abbreviations

ADC—Antibody Drug Conjugate; ABD—Albumin Binding Domain; CAR-T—Chimeric Antigen Receptor-T Cell; CDR—Complementarity Determining Region; CMC—Chemistry, Manufacturing and Controls; COMP—Cartilage Oligomeric Matrix Protein DAR—drug to antibody ratio; DMD—Duchenne Muscular Dystrophy; ECM—extracellular matrix; EGFR—endothelial growth factor receptor; FANGS—Fibronectin Antibody-mimetic Nicotinic acetylcholine receptor Generated ligands; FingRs—Fibronectin intrabodies generated with mRNA display; FN3—Fibronectin Type 3 domain; LDL—low density lipoprotein; FRET—Forster Resonance Energy Transfer; PCSK9—Proprotein convertase subtilisin kexin 9; PD-L1—programmed-cell death ligand 1; PEG—polyethylene glycol; PET—positron emission tomography; TNF—Tumour Necrosis Factor; TRAILR2—TNF-related apoptosis-inducing ligand receptor 2; VEGFR2—vascular endothelial growth factor receptor-2; VHL—Von Hippel–Lindau oncoprotein suppressor.
